# CD38 Antibodies in Multiple Myeloma: Mechanisms of Action and Modes of Resistance

**DOI:** 10.3389/fimmu.2018.02134

**Published:** 2018-09-20

**Authors:** Niels W.C.J. van de Donk, Saad Z. Usmani

**Affiliations:** ^1^Department of Hematology, VU University Medical Center, Amsterdam, Netherlands; ^2^Levine Cancer Institute, Carolinas Healthcare System, Charlotte, NC, United States

**Keywords:** CD38, antibody, daratumumab, isatuximab, MOR202, TAK-079, resistance, mode of action

## Abstract

MM cells express high levels of CD38, while CD38 is expressed at relatively low levels on normal lymphoid and myeloid cells, and in some non-hematopoietic tissues. This expression profile, together with the role of CD38 in adhesion and as ectoenzyme, resulted in the development of CD38 antibodies for the treatment of multiple myeloma (MM). At this moment several CD38 antibodies are at different phases of clinical testing, with daratumumab already approved for various indications both as monotherapy and in combination with standards of care in MM. CD38 antibodies have Fc-dependent immune effector mechanisms, such as complement-dependent cytotoxicity (CDC), antibody-dependent cellular cytotoxicity (ADCC), and antibody-dependent cellular phagocytosis (ADCP). Inhibition of ectoenzymatic function and direct apoptosis induction may also contribute to the efficacy of the antibodies to kill MM cells. The CD38 antibodies also improve host-anti-tumor immunity by the elimination of regulatory T cells, regulatory B cells, and myeloid-derived suppressor cells. Mechanisms of primary and/or acquired resistance include tumor-related factors, such as reduced cell surface expression levels of the target antigen and high levels of complement inhibitors (CD55 and CD59). Differences in frequency or activity of effector cells may also contribute to differences in outcome. Furthermore, the microenvironment protects MM cells to CD38 antibody-induced ADCC by upregulation of anti-apoptotic molecules, such as survivin. Improved understanding of modes of action and mechanisms of resistance has resulted in rationally designed CD38-based combination therapies, which will contribute to further improvement in outcome of MM patients.

## Introduction

CD38 was discovered in 1980 by E.L Reinherz and S. Schlossman, and is a type II transmembrane glycoprotein. CD38 plays a role in regulation of migration, receptor-mediated adhesion by interaction with CD31 or hyaluronic acid, and signaling events (1–3). Furthermore, CD38 also has ectoenzymatic activity and is involved in the generation of nucleotide metabolites, which play a role in the control of intracellular calcium stores (4). Under normal conditions, CD38 is expressed at relatively low levels on myeloid and lymphoid cells and in some non-hematopoietic tissues (1). In contrast, normal plasma cells and multiple myeloma (MM) cells have high levels of CD38 expression, which makes CD38 an interesting target for therapeutic antibodies targeting cell surface molecules in MM.

Currently, daratumumab (fully human; Janssen Pharmaceuticals) is the first CD38-targeting antibody, which is approved as single agent and in combination with several standards of care in MM (4). Additional CD38 antibodies that are under clinical evaluation include isatuximab (chimeric; Sanofi), MOR202 (fully human; Morphosys), and TAK-079 (fully human; Takeda) (5). CD38 antibodies are not only evaluated in relapsed/refractory MM, but also in patients with newly diagnosed MM (6). Furthermore, various preclinical studies, case reports, and clinical trials have already demonstrated promising results of CD38 antibodies in other malignancies such NK/T cell lymphoma, T-cell acute lymphoblastic leukemia, and immunoglobulin light-chain amyloidosis (7–11).

Although, immunotherapy with CD38-targeting antibodies is an attractive approach because of a favorable toxicity profile and high activity of CD38 antibodies alone or in combination with standards of care, there is substantial heterogeneity in quality and duration of response among patients. In this review, we will first describe the different modes of action of CD38 antibodies: Fc-dependent immune effector mechanisms, direct effects, and immunomodulatory effects. This is followed by a discussion of several host- and tumor-related factors that influence daratumumab efficacy. We will also discuss which mechanisms contribute to the development of acquired resistance to CD38 antibodies. An increased understanding of mechanisms underlying variability in sensitivity or acquired resistance to CD38-targeting antibodies, may lead to new strategies to improve the effectiveness of CD38 antibody-based treatment. Our review will not discuss all details of the clinical studies which evaluated CD38 antibodies, and for this topic we refer to several excellent and recently published reviews (5, 12–14).

## Mechanism of action of CD38-targeting antibodies

### Classic FC-dependent immune effector mechanisms

CD38 antibodies kill tumor cells via Fc-dependent immune effector mechanisms including complement-dependent cytoxicity (CDC), antibody-dependent cell-mediated cytotoxicity (ADCC), antibody-dependent cellular phagocytosis (ADCP), and apoptosis upon secondary cross-linking (4, 5, 15). ADCC, ADCP, and crosslinking, are dependent on the interaction of the Fc region of the antibody with Fcγ receptors (FcγRs) expressed on immune effector cells. Importantly, the CD38-targeting antibodies differ with respect to their potency to induce CDC, ADCC, ADCP, or apoptosis upon secondary cross-linking (16). This may be explained in part by unique epitopes of the different CD38 antibodies.

#### ADCC

Therapeutic antibody-mediated ADCC results in lysis of antibody-coated tumor cells by effector cells. NK-cells play a critical role in ADCC mediated by therapeutic antibodies. Indeed, depletion of NK-cells markedly reduced the capacity of daratumumab to kill MM cells via ADCC (17). Upon the binding of FcγRs to the Fc tail of the CD38-targeting antibody, NK-cells release toxic proteins including granzymes and perforins, which will kill the target cells (18). In addition, macrophages, neutrophils, eosinophils, and γδ T-cells have also been shown to induce ADCC against tumor cells coated with a therapeutic antibody (19, 20), but their role in CD38 antibody-induced ADCC is currently unknown and requires further investigations.

#### ADCP

In the process of ADCP, phagocytosis of antibody-opsonized tumor cells occurs via binding of FcγRs (such as FcγRIIA and FcγRIIIA), which are present on monocytes and macrophages. Phagocytosis contributes to the anti-tumor activity of CD38-targeting antibodies (16, 21). Interestingly, individual macrophages have the ability to quickly and sequentially engulf multiple daratumumab-coated tumor cells, indicating that ADCP is an efficient killing mechanism of daratumumab (21).

Uptake of antibody-opsonized cancer cells by antigen-presenting cells, such as macrophages and dendritic cells may also lead to enhanced antigen presentation, which may contribute to the development of tumor antigen-specific CD4^+^ and CD8^+^ T-cell immune responses (22, 23). This has been demonstrated for several therapeutic antibodies (24), but additional investigations are required to analyze to what extent FcγR-mediated enhancement of antigen presentation contributes to the anti-MM activity of CD38-targeting antibodies.

#### CDC

Binding of C1q to the Fc tail of the therapeutic antibody initiates the complement cascade, ultimately resulting in the generation of the membrane attack complex (MAC) and subsequently permeabilization of the cell membrane (25, 26). Deposition of complement components, such as C3b, on the surface of the target cell, is also the consequence of complement activation. These deposited complement components interact with complement receptors on phagocytic cells resulting in the engulfment of the tumor cells. In addition, complement activation also leads to generation of C3a and C5a. C5a increases expression of activating FcγRs, while at the same time reducing inhibitory FcγRs, which leads to enhanced phagocytosis capacity of effector cells. C3a and C5a also recruit immune cells to the tumor. Altogether, this indicates that there may be synergy between complement and the FcγR system in eliminating tumor cells (27, 28).

Daratumumab is the most effective inducer of CDC of all currently available CD38 antibodies (4). Indeed, daratumumab was selected from a panel of 42 antibodies based on its ability to strongly induce CDC (29).

### Direct effects

In an antibody screen, isatuximab was selected for further evaluation based on its ability to directly trigger MM cell death in the absence of cross-linking agents and independently of effector cells, even in cells harboring *p53* mutations (30, 31). These direct effects are independent of Fc fragment binding to Fc receptors (30). Isatuximab-mediated MM cell death is mediated by the classical caspase-dependent apoptotic pathway, as well as the lysosomal cell death pathway, which is characterized by lysosomal enlargement, lysosomal membrane permeabilization, and cathepsin hydrolase release (30). Isatuximab induces reactive oxygen species production, which occurs downstream of lysosomal activation and contributes to MM cell death (30). Daratumumab and MOR202 lack the ability to directly induce MM cell death (16). In addition, CD38 antibodies also modulate the enzymatic activity of CD38, which may contribute to MM cell death (4, 16).

It is currently unknown whether CD38 antibodies also modulate the activity of key signal transduction pathways that regulate growth and survival, as has been described for other therapeutic antibodies, such as rituximab (32). A better understanding of these potential effects, may lead to improved CD38 antibody-based combinations.

### Immunomodulatory effects

Next to the classic Fc-dependent mechanisms of action, daratumumab has also immunomodulatory effects via the elimination of CD38-positive immune suppressor cells, such as regulatory T cells (Tregs), regulatory B cells, and myeloid-derived suppressor cells (4, 33, 34). The depletion of these suppressor cells in the bone marrow (BM) microenvironment explains the increase in T-cell numbers, T-cell clonality, as well as T-cell activity following the initiation of daratumumab treatment (33, 35). Furthermore, T-cells also contain higher levels of granzyme B after exposure to daratumumab, which indicates that they have improved killing capacity (36, 37). Altogether, this suggests that daratumumab treatment leads to an improved host-anti-tumor immune response, which may be important for sustained disease control (33, 34).

Laboratory experiments showed that isatuximab also has immunomodulatory activity, but at this moment no data are available from isatuximab-treated patients. Isatuximab inhibits the suppressive function of Tregs by reducing their numbers, decreasing immune inhibitory cytokine production including IL-10, and blocking their trafficking. This results in improved NK- and T-cell-mediated anti-tumor immune responses (38).

Interestingly, exhausted T-cells not only express high levels of well-known inhibitory receptors, such as PD-1, but also CD38 (39, 40). Recent findings suggest that the NADase activity of CD38 also contributes to the development of T-cell exhaustion via reducing nicotinamide adenine dinucleotide (NAD+) levels in T-cells, resulting in decreased Sirt1/Foxo1 activity (40). Indeed, elevated levels of NAD+ in T-cells are required for an optimal anti-tumor T-cell immune response (40). Importantly, CD38 inhibition on T-cells by anti-CD38 antibodies improved anti-tumor activity in mouse models by increasing NAD+ levels (40).

## Mechanisms of resistance

In a pooled analysis of 148 patients who received daratumumab treatment as single agent at a dose of 16 mg/kg in the first in human phase 1/2 GEN501 study (41) or in the Sirius study (42), at least partial response (PR) was achieved in 31% of the patients including at least very good partial response (VGPR) in 13.5% and complete response (CR) in 4.7% (43). These patients were heavily pretreated with a median of five prior lines of therapy with 86% double-refractory to a proteasome inhibitor and an immunomodulatory drug (IMiD) (43). The median duration of response was 7.6 months. The median progression-free survival (PFS) and median overall survival (OS) were 4.0 and 20.1 months, respectively. This indicates that daratumumab induces durable responses in heavily pretreated patients. However, the majority of the responding patients develop progressive disease during daratumumab monotherapy. In addition, more than half of the patients does not respond to single agent daratumumab. Importantly, the other CD38-targeting antibodies, isatuximab and MOR202, induce similar response rates with similar response duration, when compared to daratumumab in a heavily pretreated patient population (44–46).

To improve these results, various CD38-based combinations were evaluated. Preclinical studies showed enhanced anti-MM activity when IMiDs or proteasome inhibitors were added to CD38-targeting antibodies (17, 47). IMiDs improve CD38 antibody-mediated ADCC, ADCP, direct effects, as well-immunomodulatory activity (additional details are given below) (17, 30, 36, 48). It is currently less clear why proteasome inhibitors combine well with CD38 antibodies, but this is probably related to the pleiotropic effects of proteasome inhibitors on both the MM cells and the tumor microenvironment (49). Based on these preclinical data, CD38 antibodies were combined with several standards of care for the treatment of relapsed/refractory MM patients. Adding daratumumab to lenalidomide-dexamethasone (DRd) or bortezomib-dexamethasone (DVd), led to significant improvements in clinical outcome: higher response rate, higher frequency of minimal-residual disease negativity, and improved PFS (50, 51). Based on these results, DRd and DVd were approved by both FDA and EMA for the treatment of MM patients with at least one prior line of therapy (4). The FDA also approved daratumumab in combination with pomalidomide-dexamethasone (DPd), while in Europe the results of the phase 3 APOLLO study (DPd vs. pomalidomide-dexamethasone) are required for approval of this combination. Isatuximab and MOR202 can also be effectively combined with IMiDs and proteasome inhibitors (44, 52–54).

In the following section, we will describe what is currently known about mechanisms of primary and acquired resistance to CD38-targeting antibodies. At this time, the majority of information about modes of resistance is derived from preclinical and clinical studies which evaluated daratumumab.

### Effect of prior treatment

Daratumumab as monotherapy was tested in heavily pretreated MM patients (43), but not in untreated newly diagnosed MM patients. However, laboratory studies performed with BM aspirates from MM patients, containing tumor cells and autologous effector cells, showed that the efficacy of daratumumab to induce CDC or ADCC was very heterogeneous, but without a significant difference in ADCC or CDC between samples from patients with newly diagnosed MM or relapsed/refractory disease (55). Also, in the subgroup of patients with lenalidomide- and bortezomib- (double) refractory MM, the activity of daratumumab was comparable to that observed in samples obtained from newly diagnosed patients or relapsed/refractory patients with less prior treatments (55). Data generated from these preclinical studies indicates that resistance to steroids, anthracyclins, alkylators, IMiDs, and proteasome inhibitors is not associated with reduced sensitivity to ADCC and CDC mediated by daratumumab (55).

Daratumumab is also being evaluated in patients with intermediate-risk and high-risk smoldering MM (SMM) (56). In these patients with a premalignant asymptomatic precursor disease at high risk of progression to symptomatic disease, daratumumab was evaluated in three different treatment schedules: short (16 mg/kg; one 8-weeks cycle with daratumumab administered once weekly), intermediate (16 mg/kg, one 8-weeks cycle with daratumumab administered once weekly, followed by daratumumab once every 8 weeks during cycle 2–20), and long (16 mg/kg, one 8-weeks cycle with daratumumab once weekly, then eight infusions every 2 weeks, followed by eight infusions every 4 weeks, and then infusions every 8 weeks during cycle 8–20) (56). At least PR was achieved in 38%, 54%, and 56% and at least VGPR in 15%, 24%, and 29% in the short, intermediate, and long treatment schedules, respectively. This is a higher response rate when compared to the efficacy of daratumumab in highly pretreated MM. Possible explanations for a better response in SMM include increased genetic instability from SMM to MM, altered interactions with the BM microenvironment during disease progression, and impairment of the host immune system during evolution from SMM to MM.

Interestingly, it was recently demonstrated that reintroduction of a previously failed IMiD in daratumumab-refractory patients while continuing daratumumab as a backbone, can be active in heavily pretreated MM patients (57). Similarly, the combination of pomalidomide-dexamethasone and daratumumab induces a 33% response rate in patients previously demonstrated to be refractory to both pomalidomide and daratumumab (58). In addition, 52% of heavily-pretreated lenalidomide-refractory MM patients achieve at least PR with the combination of isatuximab plus lenalidomide-dexamethasone, which is higher than what would be expected with isatuximab as a single agent (52). Altogether, this suggests that the synergistic effects between IMiDs and daratumumab, such as enhanced NK-cell and T-cell activity, potentially overcome refractoriness to both anti-MM agents.

### Cytogenetic abnormalities

The presence of high-risk cytogenetic abnormalities, such as del(17p), *t*(4;14) and *t*(14;16) is associated with a impaired survival of MM patients. High-risk MM patients benefit from CD38 antibodies, but the poor risk cytogenetic abnormalities still have a negative impact on clinical outcome in patients treated with CD38-targeting antibodies.

Twenty percent of high-risk patients achieved at least PR in the SIRIUS study (daratumumab monotherapy), while this was 29.4% for standard-risk patients (42). Interestingly, deep sustained response with daratumumab monotherapy in a high-risk patient was associated with profound reduction in Treg frequency and T-cell expansion (59).

In the randomized phase 3 POLLUX and CASTOR studies, the addition of daratumumab to Rd or Vd markedly improved the outcome of high-risk patients, when compared to Rd or Vd only. However, poor-risk conferred by the presence of del(17p), *t*(4;14), or *t*(14;16) was not completely abrogated by adding daratumumab (60). Although overall response rates with the DPd combination were similar for MM patients with standard or high-risk disease, the median PFS was inferior in high-risk patients, when compared to standard risk patients (3.9 vs. 10.3 months), while OS was similar in both groups (61). Also high-risk patients treated with isatuximab plus lenalidomide-dexamethasone or isatuximab plus pomalidomide-dexamethasone had a lower response rate, when compared to standard-risk patients (52, 62).

It is likely that other tumor-related factors, such as mutations in oncogenes and tumor suppressor genes, and activation status of signaling pathways also contribute to the variability in response to therapy with CD38 antibodies, but this requires further investigation. A better understanding of the role of molecular and biochemical mechanisms of resistance may also contribute to new combination treatments that overcome resistance.

### CD38 target antigen

#### CD38 and primary resistance

Extent of daratumumab-associated ADCC and CDC is associated with expression levels of CD38 on the cell surface (55). Indeed, CD38-overexpressing clones were more susceptible toward ADCC and CDC, when compared to the non-transduced parental cell lines (55). There is also marked heterogeneity in intensity of CD38 expression on primary MM cells without a difference between MM cells from newly diagnosed or relapsed/refractory patients (55). Similar to the observations with cell lines, daratumumab-mediated ADCC and CDC was less effective against MM cells with low CD38 expression (55).

To further understand the heterogeneity in response, we analyzed CD38 cell surface expression levels in 102 patients, who received 16 mg/kg daratumumab as monotherapy in the GEN501 and Sirius studies to analyze the impact of CD38 expression levels on response. In this analysis, MM patients who achieved at least PR had higher baseline CD38 expression levels, when compared to patients who achieved less than PR (63). Because of the substantial overlap in CD38 expression levels between responders and non-responders, selecting patients based on CD38 expression alone does not seem warranted.

Since CD38 expression is a key determinant of susceptibility of MM cells to daratumumab-mediated ADCC and CDC, as well as clinical response, several groups are evaluating agents that increase CD38 protein levels to improve the efficacy of daratumumab. Binding of all-trans retinoic acid (ATRA) to the retinoic acid receptor affects gene expression, which includes increased expression of CD38 (64, 65). This can be explained by the presence of a retinoic acid-responsive element in the first intron of the *CD38* gene (66). Interestingly, ATRA also increased CD38 expression levels on MM cell lines and primary MM cells without having an effect on MM cell viability (55). ATRA-induced CD38 upregulation markedly enhanced daratumumb-mediated ADCC and CDC against MM cells. Furthermore, ATRA increased the activity of daratumumab in MM cells, which were resistant to daratumumab in the absence of other drugs (55). Also in a humanized mouse model, ATRA and daratumumab showed synergistic anti-MM activity (55). A clinical study is currently evaluating the value of adding ATRA to daratumumab-refractory patients. Furthermore, the histone deacetylase inhibitor panobinostat induces epigenetic modifications that lead to enhanced expression of CD38 (67). The increase in CD38 antigen density by panobinostat resulted in improved daratumumab-mediated ADCC (67).

#### CD38 and acquired resistance

There is a rapid decrease in CD38 expression levels on the MM cell surface during daratumumab-treatment (63, 68). Directly following the first datatumumab infusion an ~90% reduction in CD38 expression levels is noticed on non-depleted MM cells (68). A similar CD38 reduction is observed at the time of progression during daratumumab therapy. The reduction in CD38 cell surface expression is a transient phenomenon, because CD38 levels are restored to baseline levels on the MM cells ~6 months after the last daratumumab infusion (63). Daratumumab-mediated CD38 reduction is a general phenomenon, which is also observed on non-tumor cells, such as normal B-cells, T-cells, NK-cells and monocytes (68). Daratumumab reduces CD38 on the cell surface by several mechanisms. First, in responding patients daratumumab may select for MM cells with lower CD38 expression levels, while preferentially killing the MM cells with higher levels of CD38 (68). In addition, recent studies showed that daratumumab treatment results in the clustering of CD38 molecules into distinct polar aggregates, which can subsequently be released as tumor-derived microvesicles (69). Direct internalization may also contribute to loss of CD38. Finally, active transfer of CD38-daratumumab complexes and accompanying cell membrane from MM cells to monocytes and granulocytes also contributes to CD38 reduction (68). This process of trogocytosis is in part FcγR-dependent (68).

Reduced CD38 expression on non-depleted MM cells is associated with protection against ADCC and CDC (63, 68). Reduced daratumumab-mediated ADCC and CDC induced by CD38 loss was also observed in patients with persistent response (68). Interestingly, ATRA also increased CD38 expression, almost to pretreatment values, in these daratumumab-resistant MM cells, leading to improvements in daratumumab-mediated CDC and ADCC.

Importantly, the reduction in CD38 expression levels, which is associated with impaired classic Fc-dependent immune effector mechanisms, was similar in responding and non-responding patients (63). Indeed, CD38 expression is also reduced in patients with sustained high quality response, suggesting that CD38 reduction is not necessarily associated with escape from daratumumab-mediated killing, but indicates that the pressure to keep MM cells in a state of low CD38 expression, may also offer clinical benefit. Reduced CD38 expression may result in impaired adhesion to stromal cells via CD38-CD31 interactions leading to reduced growth and impaired protection against apoptosis (70). Moreover, daratumumab-mediated trogocytosis may also impair the ability of tumor cells to interact with the protective BM microenvironment by reducing expression of several other adhesion molecules (such as CD49d, CD56, and CD138) on MM cells (68). In addition, daratumumab-mediated reduction of CD38 on MM cells may also result in reduced generation of immunosuppressive adenosine molecules (71), and thereby an improved host-anti-tumor immune response (72–74).

### Soluble CD38 and anti-drug antibodies

Soluble CD38 may neutralize CD38-targeting antibodies and thereby have an impact on pharmacokinetic profile and response. In the GEN501 and Sirius daratumumab monotherapy studies, soluble CD38 was found in only 2 out of 110 patients (63). Both patients achieved a PR with daratumumab treatment. To the best of our knowledge, impact of soluble CD38 levels on clinical outcome was not reported in the studies with MOR202 and isatuxumab (5).

In a similar way, development of anti-drug antibodies may lead to impaired activity of CD38 antibodies. Up till now, anti-daratumumab or anti-isatuximab antibodies have not been detected (41, 42, 50, 75), while development of anti-drug antibodies is a rare event with MOR202 (76).

### CDC resistance

Several fluid phase regulators, as well as membrane-associated complement-inhibitory proteins, such as CD46, CD55 and CD59, protect healthy tissues against accidental complement attack. These complement inhibitors have also been shown to confer protection of tumor cells against several therapeutic antibodies (77–79).

In an analysis of 23 MM and lymphoma cell lines, daratumumab-sensitive cell lines had lower CD59 and CD55 expression, when compared to CDC-resistant cell lines (63). No difference was found for CD46 (63). Removal of the glycosylphosphatidylinositol-anchored CD55 and CD59 molecules from the cell surface with phospholipase-C, rendered cell lines more sensitive to daratumumab-mediated CDC. In contrast, expression levels of these complement inhibitors were not associated with extent of complement-mediated lysis of primary MM cells by daratumumab (63). Similarly, in the GEN501 and Sirius studies (MM patients treated with 16 mg/kg daratumumab as single agent), there were no differences in pretreatment expression levels of CD46, CD55 and CD59 between responding and non-responding patients (63). However, at the time of progression during daratumumab therapy, a marked increase in CD55 and CD59 was observed on both MM cells localized in the BM, as well as on circulating MM cells (63). Interestingly, in some MM tumors there are coexisting subpopulations of tumor cells with markedly different levels of CD55 and CD59 expression. During daratumumab therapy, the selective pressure resulted in selection of daratumumab-resistant MM cells with high expression of complement-inhibitory proteins (63).

ATRA improved CDC to a higher extent than ADCC, which was explained by the reduction of CD55 and CD59 by ATRA, next to its effect on CD38 expression (55). Importantly, ATRA also reduces CD55 and CD59 expression levels in MM cells obtained from patients with daratumumab-refractory disease, which together with CD38 upregulation, leads to improved daratumumab-mediated CDC (63). Although the histone deacetylase inhibitor, panobinostat, induces a marked increase in CD38 expression on MM cells, CDC was not enhanced, probably as a result of a concomitant increase in CD55 and CD59 expression (67).

### ADCC resistance

#### NK-cells

In experiments with patients' samples, daratumumab-mediated ADCC was superior in samples with a high NK-cell to MM cell ratio, when compared to samples with a low ratio (55, 80–83). Similar associations were found between efficacy of daratumumab to kill primary MM cells and frequency of activated NK-cells defined as CD3^−^/CD56^+^/CD16^+^ (55).

This indicates that agents that have the ability to induce NK-cell activation may enhance daratumumab-mediated ADCC. Indeed, IMiDs, such as lenalidomide and pomalidomide, induce NK-cell activation and synergize with daratumumab in ADCC assays (17, 47, 84). In preclinical experiments, IMiDs also improve daratumumab-mediated ADCC in case of lenalidomide-refractory MM cells, indicating that the immune system of these patients is still able to respond to the immunomodulatory effects of IMiDs (17). Similarly, lenalidomide also increases anti-MM activity of CD38-targeting antibodies in patients with lenalidomide-refractory MM (52). Blocking the three main inhibitory KIR receptors (KIR2DL1/2/3) on NK cells with the IPH2102 antibody also leads to improved NK-cell activity against tumor cells (85, 86). This monoclonal antibody also enhances the efficacy of daratumumab-induced, NK-cell-mediated ADCC via the modulation of KIR-inhibitory signaling (87). Interestingly, *KIR* and *HLA* genotypes have an impact on the clinical outcome of MM patients receiving treatment with isatuximab plus lenalidomide-dexamethasone (88).

ADCC requires activation of FcγRs, which are present on the cell surface of NK-cells. Allelic variants of FcγRs with different functionality are implicated in differential response to antibody-based therapy in lymphomas and solid tumors (89–91). The FcγRIIA-131H or FcγRIIIA-158V polymorphisms are associated with a higher affinity for IgG, when compared to their allelic counterparts (92, 93). In addition, the FcγRIIB-232T polymorphism is not able to associate with lipid rafts and thereby markedly weaker in its negative regulatory activity (93). In patients treated with daratumumab monotherapy, FcγRIIIA and FcγRIIB variants have a modest impact on response and PFS, but have no significant effect on OS (94).

Although daratumumab-mediated ADCC is enhanced by agents that increase NK-cell activity, CD38 is highly expressed on NK-cells, which explains their rapid reduction in peripheral blood and BM after infusion of daratumumab (95). This reduction in NK-cells may impair tumor cell killing (95, 96). The rapid NK-cell depletion occurs due to daratumumab-mediated NK-cell fratricide via ADCC (NK-mediated cytotoxicity against neighboring NK-cells) (96). As expected, the residual NK-cells have low CD38 cell surface expression levels (68, 96). NK-cell numbers increase again 3–6 months after the last daratumumab infusion (95). Importantly, responding and non-responding patients experience similar reductions in NK-cell frequencies. The multiple mechanisms of action of daratumumab may explain the lack of association between extent of NK-cell depletion and efficacy of treatment. In addition, no relationship was observed between PFS or occurrence of side effects including infections and maximum reduction in NK-cells (95). Outcome following daratumumab therapy may be enhanced by administration of *ex vivo* expanded NK-cells (96). In addition, pretreatment of expanded NK-cells with F(ab)2 fragments of daratumumab to avoid NK-cell fratricide may represent an alternative approach to improve daratumumab-mediated ADCC in patients. However, feasibility and efficacy of this approach should be assessed in clinical trials. At this moment there is no clinical data on NK-cell frequencies available from patients treated with isatuximab or MOR202, but in *ex vivo* assays isatuximab and, to a lesser extent, MOR202, also reduce NK-cell numbers (95).

#### Bone marrow stromal cells

It is well-known that stromal cells protect MM cells against various anti-MM drugs, such as dexamethasone, doxorubicin, melphalan, lenalidomide, and bortezomib, via soluble factors or cell adhesion (97–100). It was recently shown that stromal cells also confer protection of MM cells against daratumumab-induced ADCC (101). This protection was not mediated via alteration of target expression levels or suppression of NK cell activity, but possibly via upregulation of anti-apoptotic molecules, such as survivin and Mcl-1 (101).

### ADCP resistance

Similar to CDC and ADCC, capacity of daratumumab to induce phagocytosis is in part dependent on CD38 expression levels (21). Furthermore, in *ex vivo* experiments a high monocyte-MM cell ratio resulted in improved killing of MM cells (55). Similar to ADCC, ADCP also requires activation of the FcγR. As described in the previous section, FcγR polymorphisms have a modest impact on efficacy of CD38 antibodies to eliminate tumor cells (94). Interestingly, it was recently shown that CD47 on MM cells inhibits phagocytosis induced by CD38 antibodies via ligation to SIPRα, which is expressed on phagocytes (102). Blockade of CD47-SIPRα “don't eat me” signaling may therefore increase the clinical activity of CD38 antibodies. In addition, low-dose cyclophosphamide potentiates daratumumab-mediated ADCP via enhancing FcγR expression levels on macrophages and reducing CD47 levels on tumor cells (103, 104). IMiDs also enhance the tumoricidal activity of macrophages and promote ADCP (48). Other possible determinants of ADCP efficiency of CD38 antibodies include target cell size and shape (105, 106).

### Resistance to direct effects

Extent of isatuximb-mediated direct anti-MM activity is in part dependent on CD38 target expression levels. Indeed, CD38-overexpressing cell lines were more sensitive to the direct cytotoxic effects of isatuximab, when compared to the parental cell lines (30). IMiDs enhance the direct apoptotic effects of isatuximab (30). In this respect, pomalidomide was more potently enhancing direct cytotoxic effects than lenalidomide (30).

Acquired mechanisms of resistance to these direct effects, such as altered activity of signal transduction pathways, are currently unknown, and require further investigations.

### Resistance to immunomodulatory activity

In patients treated with daratumumab as single agent, the frequency of activated T-cells declines when patients experienced relapse (33). Future studies are needed to evaluate why the number of activated T-cells is reduced at the time of relapse. In addition, single-cell RNA sequencing in patients treated with daratumumab plus IMiD revealed that responding patients are characterized by higher CD28 expression on T cells, a significantly larger cluster of central memory T cells, and a M1 activated macrophage signature, when compared to resistant or progressing patients (107).

It is currently unknown whether tumor-associated factors, such as mutations in the antigen processing and presentation pathways, loss of neoantigen expression, or insensitivity to T-cell effector molecules are associated with primary or acquired resistance to CD38-targeting antibodies (108).

Compensatory upregulation of multiple inhibitory immune checkpoints, which is implicated in the resistance to programmed cell death-1 (PD-1) or programmed death ligand-1 (PD-L1) inhibitors, may also contribute to development of resistance to the immunomodulatory activities of CD38 antibodies (108, 109). Indeed, preclinical data suggest that immunomodulatory activity of CD38 antibodies can be enhanced by combining a CD38 antibody with a PD-1/PD-L1 inhibitor. For example, in MM, lung cancer, and colon adenocarcinoma mouse models targeting the CD38 and PD-1 pathway with the combination of a CD38 antibody and PD-1 antibody resulted in enhanced anti-tumor activity, when compared to targeting either pathway alone (110). This was accompanied by increased T-cell infiltration and T-cell activation in the tumors with combined anti-CD38 and anti-PD-1 treatment (110). In addition, another group showed that CD38 expression is increased following therapy with a PD-L1 inhibitor in a lung cancer mouse model, which was associated with impaired CD8^+^ T-cell function (111). This suggests that increased CD38 expression is a novel resistance mechanism to PD-1/PD-L1 antibody treatment. As expected, enhanced antitumor activity was observed when a CD38 antibody was combined with a PD-L1 inhibitor in this lung cancer mouse model (111).

Based on these preclinical studies, various clinical trials are evaluating whether the anti-MM activity of CD38 antibodies can be enhanced by immuno-oncology combinations with PD-1 or PD-L1 inhibitors (5). Furthermore, this antibody combination is also tested in other tumors irrespective of expression of CD38 on the tumor cells (5).

Furthermore, IMiDs not only enhance ADCC and ADCP, but also increase CD38 expression levels on Tregs, which leads to enhanced isatuximab-induced inhibition of Tregs in the presence of IMiDs (38). This indicates that IMiDS also enhance the immunomodulatory activity of CD38 antibodies.

## Conclusions and future prospects

CD38-targeting antibodies utilize multiple effector mechanisms including classic Fc-dependent immune effector mechanisms, but also the recently discovered immunomodulatory mode of action contributes to anti-tumor activity. These pleiotropic mechanisms of action explain the high activity of the CD38 antibodies as single agent in heavily pretreated MM patients.

**Figure 1:**
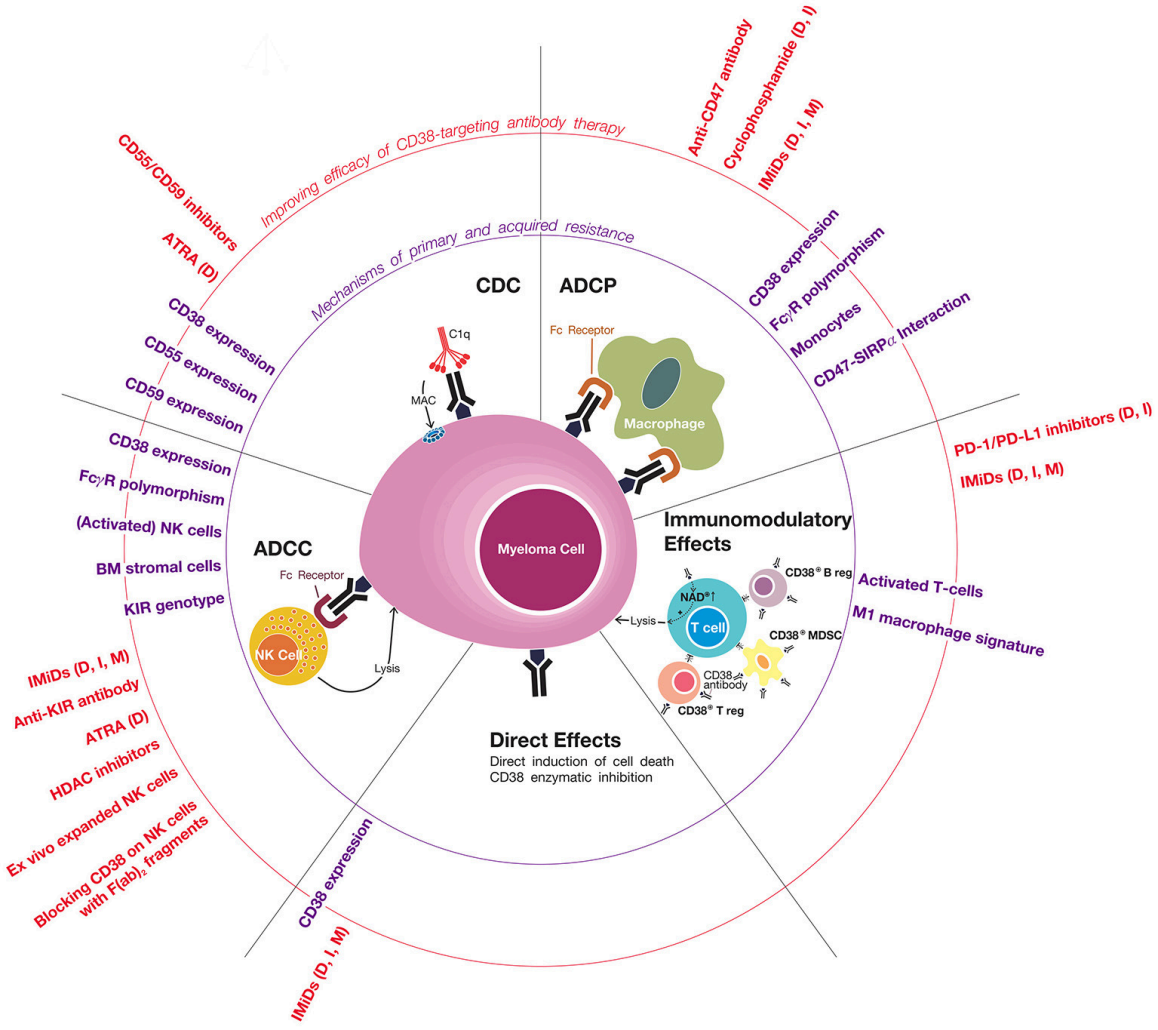
Mechanisms of primary and acquired resistance to CD38 antibodies. CD38-targeting antibodies have Fc-dependent immune effector mechanisms: complement-dependent cytoxicity (CDC), antibody-dependent cellular phagocytosis (ADCP), and antibody-dependent cell-mediated cytotoxicity (ADCC). NK cells play an important role in CD38 antibody-mediated ADCC, but the possible additional role of other effector cells, such as macrophages, neutrophils, eosinophils, and γδ T-cells, is currently unknown. Daratumumab and isatuximab also have immunomodulatory effects via the eradication of CD38-positive regulatory T-cells, regulatory B-cells, and myeloid-derived suppressor cells, which is associated with CD4^+^ and CD8^+^ T-cell expansion, and probably a better host-anti-tumor immune response. In addition, CD38 inhibition on T-cells by anti-CD38 antibodies may also contribute to improved anti-tumor activity by increasing NAD+ levels in T-cells. It is currently unknown whether MOR202 has immunomodulatory effects. In addition, isatuximab also directly induces MM cell death by both the classical caspase-dependent apoptotic pathway and lysosomal cell death pathway. Determinants and mechanisms of primary or acquired resistance to these individual modes of action are indicated (in purple), as well as strategies of how to improve these mechanisms of action in order to improve sensitivity and prevent development of resistance (indicated in red). In case the indicated agents have been tested or are being tested in a clinical trial, we added between brackets the CD38 antibody in the combination regimen (D, daratumumab; I, isatuximab; M, MOR202). General mechanisms of resistance include the presence of high-risk cytogenetic abnormailities and development of anti-drug antibodies. Of note, most data with respect to mechanisms of resistance to CD38 antibodies is derived from studies, which evaluated daratumumab. Additional studies are required for isatuximab and MOR202.

The efficacy of CD38-targeting antibody therapy can be improved by adding a partner drug with a different mode of action. Indeed, addition of an IMiD or proteasome inhibitor to a CD38 antibody leads to markedly improved outcome. Further improvement may be achieved by addition of an agent that has the ability to enhance complement activation, NK-cell-mediated ADCC, macrophage-mediated ADCP and/or host-anti-tumor T-cell immunity. Indeed, a better understanding of mechanisms that contribute to innate and acquired resistance has already resulted in the rational design of several new combinations with daratumumab, which are currently evaluated in clinical trials (Figure 1).

At the moment of development of resistance to a CD38 antibody-based treatment, an alternative treatment regimen can be selected based on several patient- and tumor-related factors, such as type of prior therapies, presence of comorbidities, and aggressiveness of relapse (112, 113). Alternatively, patients that develop resistance to a CD38 antibody may benefit from adding another drug, such as ATRA, that reverses resistance to CD38 antibodies. Several trials are currently evaluating such agents in patients who developed CD38 antibody-refractory disease (Figure 1). Another approach is to switch to a different CD38 antibody with different mode of action in case of refractoriness to CD38 antibody treatment. However, although functional differences exist between the CD38-targeting antibodies (16), it is currently unclear whether resistance to one CD38-targeting antibody confers resistance to all CD38 antibodies. A phase 1 trial is currently evaluating the value of isatuximab in daratumumab-refractory patients (NCT02514668). Alternatively, resistance to CD38 antibody-based therapy may also be reversed by adding a synergistic partner drug or changing the partner drug, while continuing the CD38 antibody (57).

Development of next generation CD38 antibodies with optimized CDC or ADCC capacity, by using new antibody engineering techniques, may also lead to more effective CD38-targeting antibodies. For example, the ability of the antibody to activate complement can be enhanced by generating targeted single amino acid changes in the Fc region of the antibody, which allows for hexamer formation upon binding to antigens on a cell (25, 26, 114). In addition, Fc glycosylation (glycoengineering) improves the affinity of the antibody for FcγRs. Indeed, the glycoengineered Fc portion of obinutuzumab enhances the binding affinity to FcγRIIIA, leading to enhanced ADCC and ADCP (115). Furthermore, bispecific antibodies that simultaneously bind to two distinct targets (epitopes on two distinct proteins or two epitopes on a single protein) may offer therapeutic benefit. In this respect, a CD38xCD3 bispecific antibody has been shown to stimulate T-cell-mediated killing of MM cells (116). Moreover, a CD38xCD59 bispecific antibody may have increased CDC activity by simultaneously targeting CD38 and neutralizing CD59 (117).

In conclusion, an increased understanding of host- and tumor-related features that underlie differential therapeutic efficacy and contribute to resistance toward CD38 antibodies, may lead to further optimization and individualization of treatment and a better outcome for MM patients.

## Author contributions

All authors listed have made a substantial, direct and intellectual contribution to the work, and approved it for publication.

### Conflict of interest statement

NvdD has received research support from Janssen Pharmaceuticals, AMGEN, Celgene, Novartis, and BMS, and serves in advisory boards for Janssen Pharmaceuticals, AMGEN, Celgene, BMS, Novartis, Bayer, Takeda, and Servier. SU reports consulting for Abbvie, Amgen, BMS, Celgene, Janssen, Takeda, Sanofi, and SkylineDx; speaker's fees for Amgen, Celgene, Janssen, and Takeda; and research funding from Amgen, Array Biopharma, BMS, Celgene, Janssen, Pharmacyclics, Sanofi, and Takeda. The Handling Editor declared a past co-authorship with one of the authors, NvdD.
